# A Novel, Broad-Spectrum, Virulent Bacteriophage Targeting *Yersinia pestis* Isolated from the Soil of Wild Rodent Nests in Yunnan Province, China

**DOI:** 10.3390/pathogens14121195

**Published:** 2025-11-24

**Authors:** Ying Long, Youhong Zhong, Pan Liu, Chunpeng Mao, Haipeng Zhang, Liyuan Shi, Shaogui Zi, Xinyu Qin, Zongti Shao, Rongji Cao, Hongbaiyu Liu, Qingwen Gao, Ling Yang, Yuming Chen, Yuanying Shen, Peng Wang

**Affiliations:** 1 Department of Medical Microbiology and Immunology, School of Basic Medical Sciences, Dali University, Dali 671003, China; 15825038684@163.com (Y.L.);; 2 Yunnan Key Laboratory for Zoonosis Control and Prevention, Yunnan Institute for Endemic Disease Control and Prevention, Dali 671000, China; zyhong520@126.com (Y.Z.); liup0506@163.com (P.L.); 18214558387@163.com (C.M.); m13529650205@163.com (H.Z.); 15087261364@163.com (L.S.);; 3 School of Public Health, Kunming Medical University, Kunming 650102, China; 4 School of Public Health, Dali University, Dali 671003, China; 5 Yunnan Pu’er Medical School, Pu’er 665099, China

**Keywords:** broad-spectrum activity, *Yersinia pestis*, genomic characteristics, novel bacteriophage, strong lytic activity

## Abstract

As promising biological tools, bacteriophages offer broad potential applications in disease diagnosis, treatment, and food safety. This study is the first to isolate a novel bacteriophage, designated vB_YpP_JC53 (abbreviated JC53), from the soil of wild rodent nests in plague-endemic areas of Yunnan Province. This bacteriophage is a T7-like phage that has the broadest host range among all T7-like phages discovered to date and remains stable under varying temperature and pH conditions. Comparative genomic analysis through NCBI revealed that the nucleotide sequence of phage JC53 shares 94.98% homology (95% coverage) with phage PSTCR2, a member of the *Solymavirus* genus, while exhibiting substantially lower similarity to known Yersinia phages. Further phylogenetic and collinearity analyses demonstrate that JC53 represents an evolutionarily distinct lineage, clearly diverging from Yersinia-infecting, T7-like, and Shigella phages, suggesting the emergence of a novel evolutionary branch. Its low ANI values relative to Yersinia phages and mosaic genome organization indicate a complex evolutionary origin, reflecting the extensive diversity of environmental phage populations. Collectively, these findings support the designation of JC53 as a novel Yersinia phage. Genome sequencing revealed that JC53 has a genome size of 39,415 bp, with a total of 52 predicted open reading frames. The broad bacteriophage spectrum of JC53 challenges the long-standing perception that T4-like bacteriophages primarily depend on a wide host range. These findings suggest that, within plague foci, JC53 may maintain ecological fitness by targeting other bacteria rather than strictly relying on *Yersinia pestis*. As a result, JC53 holds potential as an ecological control agent with the potential to suppress plague transmission by regulating the microbial community structure within foci.

## 1. Introduction

Plague is a highly virulent zoonotic disease caused by the bacterium *Yersinia pestis*. Owing to its tremendous contagiousness, rapid transmission, and consistently high mortality rates, plague has been designated by the World Health Organization as a quarantinable infectious disease requiring priority prevention and control measures [1]. This pathogen has caused three catastrophic pandemics in human history, resulting in the deaths of hundreds of millions of people [2]. Its persistence in natural reservoirs renders recurrent outbreaks a continuing major public health threat. Research has shown that *Yersinia pestis* possesses remarkable environmental adaptability and is capable of infecting more than 200 mammalian species, persisting in soil for prolonged periods while retaining its pathogenic potential [3,4]. This broad host range and environmental persistence present substantial challenges for its control and prevention. Although modern public health measures and antibiotics have significantly reduced large-scale outbreaks, natural foci of *Yersinia pestis* persist worldwide—spanning the Americas, Asia, and Africa—and continue to pose a threat to human health. More concerning is the emergence of antibiotic-resistant strains, such as those identified in Madagascan bubonic plague patients, who exhibit resistance to first-line antibiotics currently used for plague treatment and prevention. This resistance, driven by plasmid or rpsL gene mutations, undermines conventional therapeutic strategies [5,6]. Moreover, there is currently no available plague vaccine available for public use, and preventive measures for healthcare workers and researchers remain problematic during outbreaks. These challenges highlight the urgent need for the development of novel antimicrobial agents [7,8].

As natural predators of bacteria, bacteriophages offer distinct advantages in the control of plague. Numerous studies on the bacteriophage-mediated lysis of *Yersinia pestis* are currently being conducted at scientific research centers worldwide. Multiple global research institutions are actively engaged in exploring bacteriophages targeting *Yersinia pestis*. Currently, there are four major categories of important bacteriophages. (1) Highly active diagnostic phages, including PhiA1122 and Pokrovskaya (from the *Podoviridae* family), which exhibit broad-spectrum lytic activity but show cross-reactivity with *Pseudonocardia yersiniae*. Among these, PhiA1122 is recommended by the U.S. CDC as the standard diagnostic tool for plague, while China primarily uses Yep-phi as its gold-standard diagnostic phage [9]. (2) The highly specific phage L-413C (mild type) primarily targets *Yersinia pestis*, as well as several other mild-type P2 phages, including vB_YpM_22, vB_YpM_46, and vB_YpM_50 [10,11]. (3) Cross-species lytic phages: Five phages isolated from other hosts in the Eliava IBMV collection (*S. sonnei*, *S. typhimurium*, and *S. enterica*) exhibit lytic activity against *Yersinia pestis* [12]. (4) Several T4-like bacteriophages, including YpsP-PST [13], fPS-2, fPS-65, fPS-90 [14], and JC221 [15], are capable of infecting *Yersinia pestis*. Genome studies have shown that phages from the Podoviridae family (such as Berlin, Yepe2, Yep-phi, YpP-R, YpP-G, YpsP-G, and PhiA1122) are characterized by small genomes, rapid replication rates, and short lytic cycles. Morphologically, these phages belong to the T7-like phage group [16]. However, these phages exhibit a narrow lytic spectrum, typically targeting only specific strains of *Yersinia pestis*. As a result, they are primarily utilized as diagnostic tools for plague and are not easily applicable as probiotics for practical prevention and control. Phage therapy effectively addresses the issue of antibiotic resistance and has been successfully applied for bacterial disinfection in the food, environmental, and medical fields [17,18]. Research on phages targeting *Yersinia pestis*, a broad-spectrum bacteriophage with strong lytic activity, has also demonstrated its potential for plague prevention, control, and treatment.

Currently, bacteriophages targeting *Yersinia pestis* (plague phages) are isolated predominantly from the intestinal tracts of reservoir hosts such as rodents and marmots, whereas isolation from environmental matrices within natural foci (e.g., sewage and soil) remains rare. Consequently, the discovery of novel plague phages with broad host ranges and high lytic efficiency is needed. This study aims, for the first time, to isolate and characterize a novel *Yersinia pestis* bacteriophage, vB_YpPJC53, from the soil of wild rodent nests in Yunnan Province, a natural plague focus. This phage displays potent lytic activity and a broad host range, with morphological features consistent with those of members of the family Podoviridae. Its preliminary biological properties were evaluated through host range assays, one-step growth curve analysis, and stability testing under various stress conditions. The genome of vB_YpPJC53 was annotated and subjected to comparative analysis, and its phylogenetic relationship with all Yersinia-infecting phages archived in the NCBI database was elucidated. The emergence of this phage challenges the long-standing reliance on broad host-range T4-like phages as the primary agents for controlling plague-associated microorganisms in endemic areas, thereby providing a new potential strategy for plague prevention and control. These findings also underscore the necessity of further investigating certain types of phages with unique biological and therapeutic potential.

## 2. Materials and Methods

### 2.1. Bacterial Strains and Bacteriophage Culture Conditions

This study employed the *Yersinia pestis* vaccine strain EV76 as the indicator strain for phage isolation. Both this strain and the 80 additional strains used in the lysis spectrum assays, including 32 *Y. pestis* strains, 2 *Y. enterocolitica* strains, 6 *Y. pseudotuberculosis* strains, 11 *Shigella* strains, 2 *Salmonella* strains, 6 *Enterobacter* strains, 3 *Klebsiella* strains, 5 *Escherichia* strains, 2 *Acinetobacter* strains, 4 *Proteus* strains, and single representatives of *Klebsiella pneumoniae*, *Proteus mirabilis*, *Acinetobacter baumannii*, *Enterobacter cloacae*, *Streptococcus agalactiae*, *Enterococcus faecalis*, *Serratia marcescens*, *Enterobacter agglomerans*, and *Providencia* species, were preserved at the Yunnan Provincial Institute for Endemic Disease Control. All Enterobacteriaceae strains were propagated in Luria–Bertani (LB) broth and on LB agar plates. Phages, *Y. pestis*, and *Y. enterocolitica* were maintained at 28 °C, whereas the other *Enterobacteriaceae* strains were cultured at 37 °C.

### 2.2. Specimen Collection and Processing

From April 2023 to May 2024, soil samples were collected from rodent nests in areas with relatively active wild rodent plague foci in Jianchuan County and Yulong County, Yunnan Province. Nest soil was aseptically transferred into 50 mL centrifuge tubes. Each sample was then suspended in SM buffer (5.8 g/L NaCl, 2.0 g/L MgSO_4_, 50 mL/L 1 M Tris [pH 7.5], and 5 mL/L 2% gelatin) and stored at 4 °C until further analysis.

### 2.3. Phage Isolation and Purification

Two milliliters of the supernatant from the settled soil suspension was mixed with 500 μL of the host bacterium *Y. pestis* EV76 suspension and 15 mL of LB broth. The mixture was thoroughly mixed and incubated at 28 °C in a shaking incubator at 220 rpm for 18–24 h. The culture was then filtered through a 0.22 μm syringe filter. Afterward, 200 μL of the filtrate was combined with 100 μL of EV76 bacterial suspension in the logarithmic growth phase, added to LB semisolid medium and maintained at approximately 50 °C. After thorough mixing, the preparation was spread onto double-layer agar plates and incubated at 28 °C. Plaques were observed after 18–24 h. When plaques appeared, a single clear, translucent plaque with well-defined edges was selected for amplification and purification. This plaque purification procedure was repeated four to five times to obtain purified phage filtrates. The resulting phage solution was amplified and stored in 25% glycerol at 4 °C for short-term use and at −80 °C for long-term preservation.

### 2.4. Electron Microscopy of Plague Bacteriophages

The purified bacteriophage suspensions were applied to 400-mesh carbon-coated copper grids and allowed to adsorb for 1–2 min. The excess liquid was gently removed with filter paper, after which the grids were negatively stained with 2% phosphotungstic acid (pH 6.5) for 1 min and air-dried. Phage morphology was examined and imaged at appropriate magnification using a Hitachi HT7700 transmission electron microscope (Hitachi, Tokyo, Japan) operated at 80 kV. The dimensions of three individual viral particles were measured, and the mean values were calculated.

### 2.5. Determination of Phage Biological Characteristics

#### 2.5.1. Temperature and pH Stability Assays

Purified phage suspensions with a titer of 10^8^ PFU/mL were incubated in water baths at 4 °C, 21 °C, 28 °C, 37 °C, 50 °C, 60 °C, and 70 °C; after 1 h of incubation, the phage titer was determined using the spot assay method, with three replicates performed under each temperature condition. For pH stability testing, buffers with pH values ranging from 0 to 14 were prepared. The phage suspension was incubated in each buffer for 1 h, after which phage activity was evaluated using the spot test. All experiments were conducted in triplicate.

#### 2.5.2. Phage Ethanol Tolerance and UV Sensitivity Assays

A bacteriophage suspension (10^8^ PFU/mL) was exposed to a 15 cm ultraviolet lamp for 0–30 min, and samples were collected at 3 min intervals. In parallel, aliquots were treated with ethanol at concentrations of 10%, 25%, 50%, 75%, and 95% (*v*/*v*) for 1 h. Phage activity (titer) was assessed using a spot-drop assay. All experiments were performed in triplicate [15].

#### 2.5.3. Determination of the Optimal Multiplicity of Infection and Construction of the One-Step Growth Curve for the Bacteriophage

EV76 bacterial cultures in the logarithmic growth phase were cocultured with the phage suspension at multiplicities of infection (MOIs) of 0.0001, 0.001, 0.01, 0.1, 1, and 10 for 6 h. Phage activity was measured using the spot inoculation (spot assay) method. All experiments were performed in triplicate. The one-step growth curve of the bacteriophage was constructed following a previously described method, with minor modifications [19]. The phage suspension was mixed with an EV76 bacterial culture at a multiplicity of infection (MOI) of 0.001 and incubated at 28 °C in a shaking incubator for 15 min. The mixture was centrifuged, and the supernatant was discarded. The resulting pellet was subsequently washed 3–4 times with LB broth, after which the supernatant was discarded. Finally, the pellet was resuspended in 10 mL of LB broth. A sample was collected at time point 0, after which the culture was immediately transferred to a shaking incubator at 28 °C for continued growth. Samples were collected at regular intervals over a 130 min period. After centrifugation to remove the supernatant, phage activity was determined using a spot assay. The burst size was calculated as the ratio of the final phage titer to the initial bacterial cell count at the time of infection. All experiments were performed in triplicate [20].

### 2.6. Determination of the Phage Host Range

The lytic activity of the bacteriophage JC53 against 81 bacterial strains was assessed using a spot test and the double-layer agar method. Lysis was evaluated at 21 °C, 28 °C, and 37 °C.

### 2.7. Phage Genome Isolation and Analysis

Phage genomic DNA was extracted using the Phage Genomic DNA Extraction Kit (Abigen, Beijing, China). Whole-genome sequencing was performed by Beijing Annohe Gene Sequencing Co., Ltd. (Beijing China), using the Illumina HiSeq platform. The phage genome sequences were aligned using NCBI BLAST+ software (version 2.16.0). The functions of the predicted genes were further annotated, and potential genes within the phage genome were identified using RAST analysis [21]. A genome-wide map of functionally annotated genes was generated using Proksee, and a comparative genetic map was constructed using Easyfig 2.2.5 software [22]. FastANI software (version 1.32) performs pairwise genome comparisons at the nucleotide level to calculate the average nucleotide identity (ANI) of the phages [23]. A whole-genome phylogenetic tree was constructed using the bioinformatics software Orthofinder (version 2.5.4), while a protein phylogenetic tree was generated using MEGA11. The phage genome was analyzed for virulence and antibiotic resistance genes using the Comprehensive Antibiotic Resistance Database (https://www.mcmaster.ca/) and the Virulence Factors of Bacterial Pathogens Database (VFDB; https://www.mcmaster.ca/).

## 3. Results

### 3.1. Isolation and Morphology of the vB_YpP_JC53 Bacteriophage

**Figure 1:**
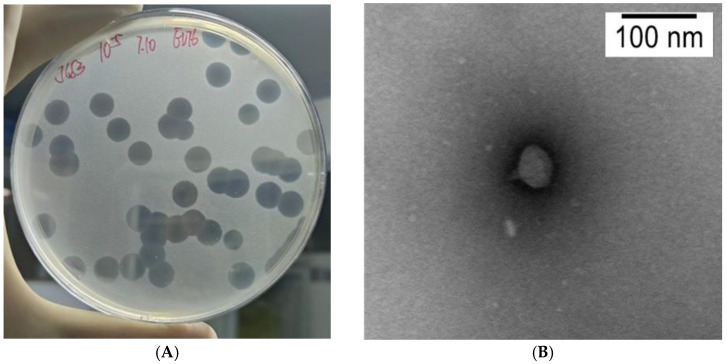
(**A**) Plaque morphology of the JC53 bacteriophage, showing distinct and transparent plaques. (**B**) Transmission electron micrograph ofJC53, showing the typical T7-like bacteriophage morphology.

A plague bacteriophage strain was isolated from soil samples collected from wild rat nests in Jianchuan County and designated vB_YpP_JC53. Electron microscopy revealed a head length of 45 ± 5 nm with a hexagonal structure and a tail length of 20 ± 5 nm (Figure 1B). According to the International Committee on Taxonomy of Viruses (ICTV) standards, the characteristics of JC53 are consistent with those of the self-replicating short-tailed phage family, and it belongs to the T7 phage class [24]. After multiple rounds of propagation and purification, phage vB_YpP_JC53 formed clear, transparent plaques on double-layer agar plates with a diameter of approximately 7.5 mm. The experimental results demonstrated that vB_YpP_JC53 effectively lysed EV76 at 28 °C, 21 °C, and 37 °C. Continuous observation under 28 °C culture conditions (Figure 1A) revealed that vB_YpP_JC53 exhibited a typical target-like plaque morphology, with expanding halo rings forming around the periphery of each plaque over time.

### 3.2. Biological Characteristics of vB_YpP_JC53

#### 3.2.1. Stability Analysis of vB_YpP_JC53

**Figure 2:**
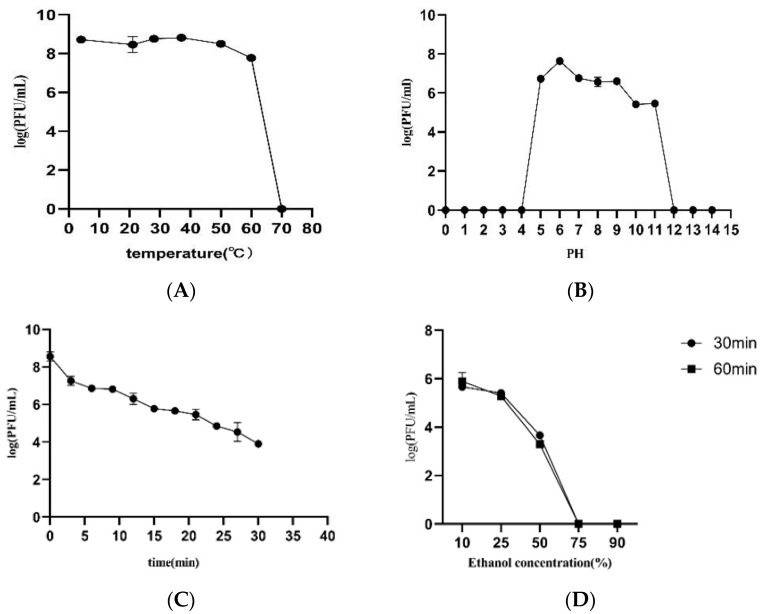

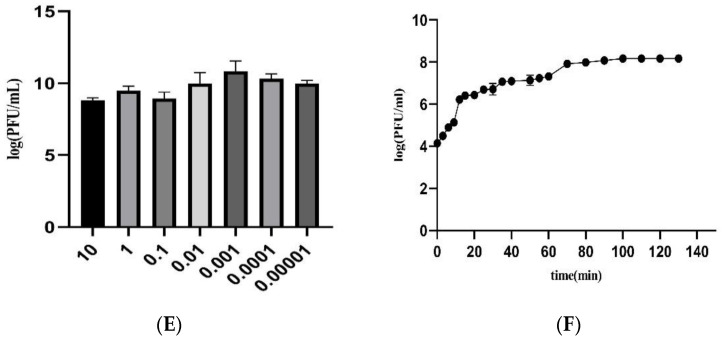
Biological characteristics of phage JC53. (**A**) Stability at different temperatures; (**B**) stability at different pH values; (**C**) sensitivity to UV irradiation; (**D**) effect of ethanol at different concentrations; (**E**) optimal multiplicity of infection (MOI); (**F**) one-step growth curve.

The exposure of phage vB_YpP_JC53 to different temperatures for 1 h revealed that its activity remained relatively stable below 60 °C, but its activity gradually decreased upon increasing temperature and was completely lost at 70 °C (Figure 2A). Compared with previously reported counterparts, the phage exhibited greater thermal tolerance. The optimal pH for vB_YpP_JC53 was determined to be 6, whereas its activity was completely abolished at pH values of ≤4 or ≥12 (Figure 2B). Upon ultraviolet irradiation, the titer of vB_YpP_JC53 decreased progressively over time, yet substantial activity remained after 0.5 h of exposure (Figure 2C). In contrast, treatment with ethanol at concentrations greater than 75% for more than 30 min effectively inactivated the phage (Figure 2D).

#### 3.2.2. Optimal Multiplicity of Infection and One-Step Growth Curve of the Bacteriophage

The EV76 bacterial suspension was maintained at a fixed concentration of 10^8^ CFU/mL, and experiments were carried out by adjusting the titer of the bacteriophage. The highest titer was observed when the bacteriophage and bacteria were mixed at a ratio of 0.001:1, corresponding to a multiplicity of infection (MOI) of 0.001 for phage vB_YpP_JC53 (Figure 2E). The one-step growth curve is shown in Figure 2F. The titer of phage vB_YpP_JC53 increased significantly after 20 min and stabilized at 80 min, with a calculated burst size of approximately 50 pfu/cell.

### 3.3. Host Range of vB_YpP_JC53

As shown in Table 1, 81 host bacteria were selected to determine the host range of vB_YpP_JC53. Among them, all 31 *Yersinia pestis* strains isolated from plague foci in house mice and wild rodents, except for EV76, were completely lysed at 21 °C, 28 °C, and 37 °C, resulting in the production of clear and transparent plaques. In addition, JC53 partially lysed *H. pseudonoculosa* serogroup II, *Shigella sonnei* serogroup I, and strain 52202 (O:2 serotype) at 21 °C and completely lysed *H. pseudonoculosa* serogroups III, IV, and VI. At 28 °C, JC53 partially lysed *H. pseudotuberculosis* serogroups I, II, and VI and completely lysed serogroups III and IV. At 37 °C, JC53 partially lysed *H. pseudotuberculosis* serogroup IV and *Shigella sonnei* serogroup II, whereas complete lysis of *H. pseudotuberculosis* serogroups I, II, III, and VI was observed. Analysis of the lysis patterns of T7-like plague bacteriophages reported to date revealed that this strain has a broad host range and can survive in plague-endemic environments even in the absence of *Yersinia pestis*.

### 3.4. Whole-Genome Characterization of the Bacteriophage vB_YpP_JC53

Through genome sequencing combined with bioinformatics analysis, we obtained the high-quality complete genome sequence of bacteriophage vB_YpP_JC53. The genome comprises linear double-stranded DNA and is 39,415 bp in length with a G + C content of 42.1%, which is lower than that of *Yersinia pestis* (47.6%). Gene annotation performed using the RAST server predicted 52 open reading frames (ORFs) within the JC53 genome, with no tRNA genes identified. All the ORFs were transcribed in the forward direction. Among these, 16 ORFs were predicted to encode functional proteins, while the remaining 36 were annotated as putative proteins. In addition, PhageLeads screening revealed no genes associated with lysogeny, antibiotic resistance, or bacterial virulence. Collectively, these findings suggest that phage JC53 is safe and has therapeutic potential [25]. Importantly, the absence of lysogeny-related genes further supports its classification as a strictly lytic phage.

**Figure 3:**
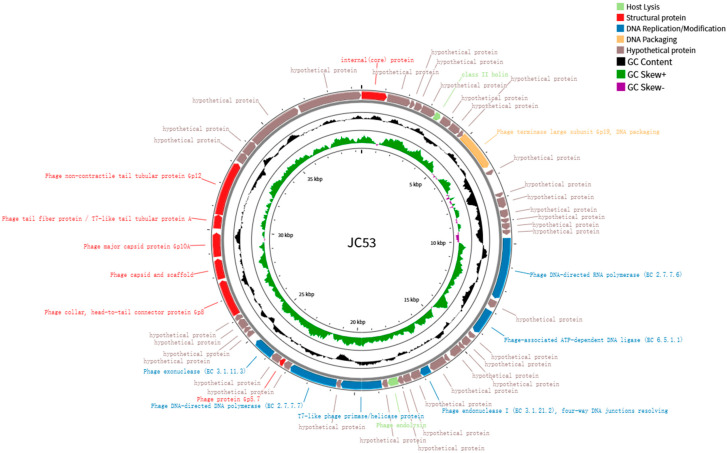
Circular genome map of bacteriophage vB_YpP_JC53. This figure illustrates the circular genomic organization of bacteriophage JC53 with corresponding functional annotations. Open reading frames (ORFs) are color-coded based on predicted functions: structural proteins (bright red), DNA replication and modification proteins (dark blue), DNA packaging proteins (orange), host lysis-associated proteins (bright green), and hypothetical proteins (light gray). The inner ring represents GC content (black) and GC skew (green for positive values, purple for negative values), highlighting the modular architecture characteristic of phage genomes.

On the basis of the functional annotation, the 16 predicted proteins of JC53 were categorized into five modules: structural, DNA packaging, DNA metabolism and replication, host lysis, and hypothetical proteins (Figure 3). These proteins cooperate through functional complementarity and precise coordination to facilitate efficient and accurate DNA packaging, ultimately ensuring the complete incorporation of viral DNA into the capsid [26]. In JC53, proteins involved in DNA packaging include the major subunit of terminase. Six ORFs encode structural proteins such as capsid, tail, and head proteins. Another six ORFs encode proteins associated with nucleic acid metabolism and replication, including nucleic acid exonucleases, DNA polymerases, DNA helicases, nucleic acid endonucleases, ATP-dependent DNA ligases, and DNA-dependent RNA polymerases. Notably, no proteins related to the lytic module were predicted in this phage strain. However, lysin (ORF31) and perforin (ORF6) were identified as participating in host lysis.

### 3.5. Genomic Synteny Analysis of the Bacteriophage vB_YpP_JC53

**Figure 4:**
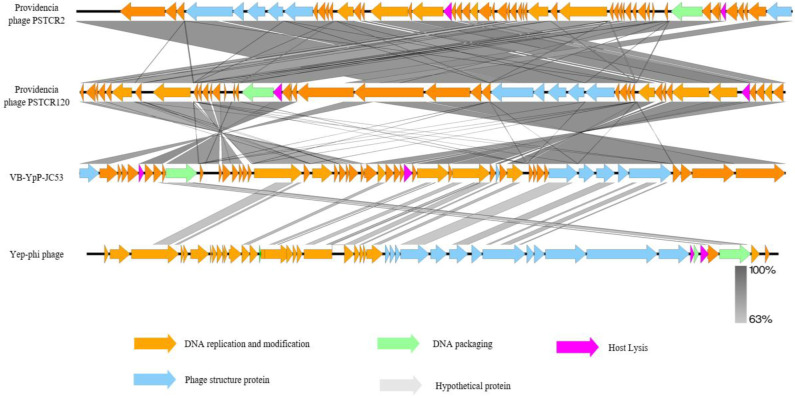
Synteny analysis of the genome of bacteriophage vB_YpP_JC53. Comparative genome synteny analysis of Providencia phages PSTCR2 and PSTCR120, Yersinia phage vB_YpP_JC53, and the T7-like phage Yep-phi. Arrows represent predicted open reading frames (ORFs), color-coded according to their functional categories: DNA replication and modification (orange), structural proteins (blue), DNA packaging (green), host lysis (pink), and hypothetical proteins (gray). Gray shading between genomes indicates regions of nucleotide homology, with color intensity reflecting the degree of sequence similarity (63–100%). The extensive synteny between JC53 and Providencia phage PSTCR2 suggests a close evolutionary relationship, while the divergence from the T7-like phage Yep-phi indicates that JC53 represents an independent evolutionary lineage within the Podoviridae family.

Transmission electron microscopy revealed that phage JC53 exhibits typical T7-like morphological characteristics. Based on genome alignment using NCBI data, Providencia phages PSTCR2 and PSTCR120 were found to share high nucleotide identity with JC53. To further elucidate their genomic architecture and evolutionary relationships, a comparative genome synteny analysis was conducted among Providencia phages PSTCR2, PSTCR120, JC53, and the T7-like representative phage Yep-phi (Figure 4). The results demonstrated that JC53 possesses distinct genomic features and exhibits extensive but differentiated sequence synteny with PSTCR2 and PSTCR120. The DNA replication and modification modules (orange) are highly conserved among the three genomes, reflecting the core stability of replication-related functions. In contrast, the structural protein modules (blue), DNA packaging modules (green), and host lysis modules (pink) show functional conservation but notable sequence divergence. The gray regions representing putative proteins correspond to genes of unknown function, which may contribute to host specificity or environmental adaptability.

The high density and continuity of gray synteny connections between JC53 and Providencia phage PSTCR2 indicate a close evolutionary relationship, whereas the divergent regions compared with the T7-like phage Yep-phi suggest an independent evolutionary trajectory. Collectively, these findings highlight JC53’s unique evolutionary characteristics within the Podoviridae family, its conserved replication machinery shared with Providencia phages, and its potential genomic adaptations to specific host environments.

### 3.6. Homology and ANI

To elucidate the genomic relationship of JC53 within the Yersinia phage community, its nucleotide sequence was aligned against 155 characterized Yersinia phages available in the NCBI database. The analysis revealed consistently low average nucleotide identity (ANI) values across all comparisons (Figure S1). Notably, JC53 did not cluster with any previously described Yersinia phages, even under relaxed ANI thresholds, indicating extremely low sequence similarity to known phages. The marked nucleotide divergence and absence of clustering suggest that JC53 represents a genetically distinct lineage with an independent evolutionary origin. Collectively, these findings support the designation of JC53 as a novel Yersinia phage at the whole-genome level.

### 3.7. Phylogenetic Analysis of the Bacteriophage vB_YpP_JC53

A phylogenetic tree was constructed based on the whole-genome sequences of JC53, 155 Yersiniaphages, and 22 Shigella phages retrieved from the NCBI database (Figure S2). The phylogenetic analysis revealed that phage JC53 is evolutionarily distinct, displaying clear divergence from both Yersinia and Shigella phages. These results indicate that JC53 represents a novel and genetically independent phage strain

## 4. Discussion

The head of phage vB_YpP_JC53 was examined in this study and displays an icosahedral morphology, while its tail is relatively short and lacks retraction capability. These structural features are characteristic of T7-like phages; thus, vB_YpP_JC53 has been classified primarily as a T7-like phage [27]. Comparative genomic analysis with other members of the genus Solymavirus revealed that vB_YpP_JC53 shares more than 95% sequence homology with the Solymavirus phage PSTCR2. In accordance with the classification criteria, phage genomes whose sequence similarity is greater than 50% are assigned to the same genus [28]. JC53 rapidly lyses *Y. pestis* strain EV76 at 28 °C within 4–5 h without generating flocculent bacterial debris, indicating high lytic efficiency. JC53 produces completely transparent plaques with distinct halos on EV76 colonies. As the incubation progresses, the plaque size stabilizes, whereas the halo regions continue to expand. The presence of halos indicates that this plague phage not only possesses strong lytic activity but also the formation of halos suggests that JC53 may exhibit polysaccharide depolymerase activity, a property commonly associated with enzymes that degrade bacterial surface polysaccharides. Genome annotation of JC53 identified structural protein modules—such as a putative phage tail fiber protein and a T7-like tail tubular protein A—that may contain depolymerase-related domains. These proteins are presumed to assist phage adsorption and infection by degrading bacterial surface polysaccharides, disrupt biofilms, increase host susceptibility to antibiotics, and participate in bacterial cell wall degradation. Collectively, these features indicate that the putative polysaccharide depolymerase likely plays an important role in the infection process of JC53 [29,30].

This bacteriophage efficiently lyses 32 strains of *Yersinia pestis* from diverse epidemic foci, including isolates from both domestic and wild rodents, and exhibits lytic activity against certain strains of *Yersinia pseudotuberculosis* and *Shigella sonnei* under different temperature conditions. Notably, its activity against *Y. pseudotuberculosis* is temperature dependent: it completely lyses Type III, IV, and VI strains at 21 °C and 28 °C while maintaining full lytic capacity against Type I, II, III, and VI strains at 37 °C. This temperature-dependent lysis pattern may be associated with variations in host receptor expression or conformational changes in phage attachment proteins. In addition, its ability to lyse certain Shigella species suggests possible recognition of conserved bacterial surface structures, such as lipopolysaccharides or outer membrane proteins. This property warrants further investigation to clarify the receptor-binding mechanism of JC53. The plague phages currently reported in the NCBI database and published literature—such as Berlin, Yepe2, YpP-G, Yep-Phi, and phiA1122—are classified within the family Podoviridae and are predominantly T7-like phages. These phages display highly specific lytic activity against *Yersinia pestis*. In 2023, Tamar Suladze et al. were unable to isolate a phage specific to *Yersinia pestis*; however, they unexpectedly obtained two phages from the Podoviridae family, namely YpYeO9 and YpEc11. Although these phages are capable of cross-lysing *Yersinia pestis,* their natural hosts are other bacterial species, specifically *Yersinia enterocolitica* and *Escherichia coli*, against which they exhibit significantly greater lytic activity [12]. Chinese researchers isolated a virulent plague bacteriophage, vB_YpP-YepMm (YepMm), from the bone marrow of naturally deceased marmots on the Qinghai–Tibet Plateau for the first time. The morphology of this bacteriophage is consistent with the characteristics of the family Podoviridae, and its genome shows 99.9% identity with that of Yep-phi [31]. This phage is capable of lysing *Yersinia pestis*, whereas most T7-like plague bacteriophages identified to date exhibit relatively narrow host ranges compared with that of the bacteriophage JC53. JC53 not only has a broader host range but also has no significant similarity to any existing diagnostic plague bacteriophages according to comparative genomic analysis. Its nucleotide identity and coverage remain low, placing it on a distinct phylogenetic branch. This genetic uniqueness greatly reduces the risk of misidentification or cross-reactivity arising from genomic homologous recombination during practical applications, thereby providing a key advantage for the use of JC53 as a stable and reliable agent for biological identification and control. In addition, JC53 retains favorable traits, such as a compact genome, rapid proliferation, and a short lytic cycle, further supporting its potential for the rapid field detection and control of pathogenic Yersinia species.

Notably, genomic alignment revealed more than 95% sequence homology between JC53 and PSTCR2, a phage of the genus *Solymavirus*. On the basis of these observations, lysis assays were performed against *Providencia stellae* and *Providencia stellae* var. *alkaligenis*. The results revealed that JC53 was unable to lyse *Providencia rettgeri*. A possible explanation for this result is that the OmpF and Ail proteins of *Yersinia pestis* facilitate the adsorption of the T7-related phage Yep-phi [32], whereas structural differences in the homologous OmpF protein of *P. rettgeri* may prevent genome injection after adsorption. Additionally, differences in LPS and capsular structures influence lysis efficiency: the rough-type LPS of *Y. pestis* EV76 more readily exposes receptors, whereas the LPS, O antigen, or capsule of *P. rettgeri* may physically shield outer membrane proteins, thereby hindering phage penetration or DNA injection [33]. Furthermore, *P. rettgeri* may employ multiple defense mechanisms, such as robust restriction–modification (R–M) systems or CRISPR—Cas immunity, which directly degrade phage DNA or provide immune memory against infection [34,35]. Despite the high degree of genomic similarity (94.98%) between JC53 and PSTCR2, the ~5% sequence divergence in key genes (e.g., those encoding endolysins) may result in functional deficiencies that impede completion of the lytic cycle.

The JC53 phage genome is packaged in a linear form with a G + C content of 42.1%, which is lower than that of *Yersinia pestis* (47.6%). This difference in GC content may reflect distinct selective pressures and mutation patterns during evolution [36]. GC content is associated with environmental adaptability, as a higher GC content typically improves DNA thermal stability. The lower GC content of JC53 compared with that of *Y. pestis* may suggest a unique adaptive strategy within the microenvironment of *Y. pestis* [37]. Long-term symbiotic phage–host systems typically exhibit GC content convergence. However, the difference in the GC content between the JC53 phage and *Yersinia pestis* suggests that the host range of JC53 is broader, a conclusion further validated by experimental evidence [38,39]. GC content influences DNA stability, transcription, and translation efficiency. The lower GC content of JC53 may differentiate it from *Y. pestis* in terms of its genomic structure, gene expression, and host interactions, thus affecting its relationship with the host bacterium [40]. Additionally, as a linear genome with forward transcription, JC53 possesses unique gene expression regulatory mechanisms. Phylogenetic tree and synteny analyses place JC53 in a more isolated position, indicating significant evolutionary divergence from both Yersinia phage 155 and typical T7-like phages and 22 Shigella phages. These findings suggest that JC53 may represent a novel evolutionary branch, potentially harboring unique functional modules (such as lytic enzymes or DNA polymerases) that drive host adaptation and diversification. Further comparative genomic analysis will help elucidate its synteny relationships with known phages and the variation patterns of key genes. On the basis of ANI analysis, the low ANI value of JC53 may reflect the diversity of phages in the environment, likely due to the mosaic nature of phage genomes, which are assembled from genes of diverse origins. JC53 has likely undergone strong selective pressure and horizontal gene transfer during genomic evolution [41]. Recent advances in viral metagenomics have enabled the rapid discovery of novel phage catalogs across various environments, revealing the diversity of phage genomes. These findings suggest that JC53 may represent a new phage species or even a new genus [42]. The C53 bacteriophage exhibits broad-spectrum lytic activity, with particularly strong lytic effects against Yersinia pestis. Its unique biological characteristics, genomic features, and phylogenetic position suggest that it may represent a novel taxonomic unit. After thorough validation through in vivo experiments and other rigorous testing, it shows promising potential for applications in plague prevention and control, as well as bacteriophage therapy.

## 5. Conclusions

This study presents the first isolation and identification of a novel, broad-spectrum, highly lytic plague bacteriophage from rodent nests in the wild rodent plague foci of Yunnan Province. The phage genome shows high similarity (94.98% sequence identity) to that of the PSTCR2 bacteriophage but exhibits notable differences in host range and evolutionary relationships. The bacteriophage JC53 efficiently lyses *Yersinia pestis* and *Yersinia pseudotuberculosis* and partially lyses *Shigella sonnei* and *Yersinia enterocolitica* (strain 52202), demonstrating its broad host range. Its short incubation period and large burst size suggest strong proliferative capacity. Moreover, the temperature adaptability of this bacteriophage is good, increasing its potential for application in diverse environments. Phylogenetic analysis placed this phage in an independent clade with low collinearity with existing plague diagnostic phages, indicating a unique evolutionary pathway and infection mechanism. Given its broad host range, potent lytic activity, and excellent environmental adaptability, this bacteriophage holds significant potential for use in biological control of plague. Through comprehensive studies, including in vivo experiments, it could be employed for plague prevention and control in natural foci. Future research should focus on optimizing phage cocktail therapies, expanding their host range through genetic engineering, and assessing their bacteriostatic efficacy in real-world settings to facilitate practical implementation.

## Figures and Tables

**Table 1:** Host Range of Bacteriophage vB_YpP_JC53.

(No.)	Species of Stains	Name of Stains	JC53		
			21 °C	28 °C	37 °C
1	*Y. pestis* (biovar 1.1N3) from wild rodent plague foci	YN1060	++	++	++
2		YN1327	++	++	++
3		YN1036	++	++	++
4		YN777	++	++	++
5		YN459	++	++	++
6		YN462	++	++	++
7		YN1075	++	++	++
8		YN1323	++	++	++
9		YN400	++	++	++
10		YN480	++	++	++
11		YN794	++	++	++
12		YN466	++	++	++
13		YN414	++	++	++
14		YN1077	++	++	++
15		YN402	++	++	++
16	*Y. pestis* (biovar 1.ORI2) from house mouse plague foci	YN1938	++	++	++
17		YN1827	++	++	++
18		YN1377	++	++	++
19		YN1788	++	++	++
20		YN2445	++	++	++
21		YN1481	++	++	++
22		YN727	++	++	++
23		YN1483	++	++	++
24		YN1394	++	++	++
25		YN1843	++	++	++
26		YN1942	++	++	++
27		YN623	++	++	++
28		YN1666	++	++	++
29		YN944	++	++	++
30		YN2403	++	++	++
31		YN404	++	++	++
32	*Yersinia pseudotuberculosis*	*Y. pseudotuberculosis* I	-	+	++
33		*Y. pseudotuberculosis* II	++	++	++
34		*Y. pseudotuberculosis* III	++	++	++
35		*Y. pseudotuberculosis* IV	++	++	+
36		*Y. pseudotuberculosis* V	-	-	-
37		*Y. pseudotuberculosis* VI	++	+	++
38	*Shigella*	*S. sonnei serotype* I	+	-	-
39		*S. sonnei serotype* II	-	-	+
40		*S. flexneri* 1a	-	-	-
41		*S. flexneri* 2a	-	-	-
42		*S. flexneri* 3a	-	-	-
43		*S. flexneri* 4a	-	-	-
44		*S. flexneri variant X*	-	-	-
45		*S. boydii*	-	-	-
46		*S. dysenteriae*	-	-	-
47		*S. dysenteriae* 1	-	-	-
48		*S. dysenteriae* 2	-	-	-
49	*Y. enterocolitica*	52202	+	-	-
50		52301	-	-	-
51	*Enterobacteriaceae*	*E. aerogenes* 11	-	-	-
52		*P. stuartii*	-	-	-
53		*P. alcalifaciens*	-	-	-
54		*E. cloacae* 138	-	-	-
55		*E. cloacae*	-	-	-
56		*E. albertii*	-	-	-
57		*E. cloacae* 18	-	-	-
58		*E. faecium* 16	-	-	-
59		*E. aerogenes* 9	-	-	-
60		*E. faecium* 5	-	-	-
61		*E. coli* 1040	-	-	-
62		*E. coli* 1095	-	-	-
63		*E. coli* 59	-	-	-
64		*E. coli* 41	-	-	-
65		*E. coli* H	-	-	-
66	*Klebsiella* spp.	*K. pneumoniae*	-	-	-
67		*K. aerogenes* 241	-	-	-
68		*K. oxytoca* 29	-	-	-
69	*γ-Proteobacteria*	*P. putida*	-	-	-
70		*S. maltophilia*	-	-	-
71		*A. hydrophila*	-	-	-
72		*P. aeruginosa*	-	-	-
73	*Acinetobacter*	*A. baumannii* 4	-	-	-
74		*A. baumannii* 33	-	-	-
75	*Pantoea*	*P*. spp. 1	-	-	-
76		*P. agglomerans* 49	-	-	-
77	*Salmonella*. spp.	*S. Typhi* 25	-	-	-
78		*S. paratyphi* B	-	-	-
79	*Serratia* spp.	*S. marcescens*	-	-	-
80	*Proteus* spp.	*P. mirabilis*	-	-	-
81	*Burkholderia* spp.	*B. cepacia*	-	-	-

Legend: Symbols indicate lysis efficiency: “++” = clear plaques (complete lysis); “+” = turbid plaques (partial or moderate lysis); “-;” = no plaques (no lysis).

## Data Availability

All data supporting this study are included in the article and its Supplementary Materials. The main genomic data (the JC53 phage sequence) presented in the study are openly available in NCBI with the accession number of BankIt3021949 JC53 PX563682.

## Supplementary Materials

The following supporting information can be downloaded at: https://www.mdpi.com/article/10.3390/pathogens14121195/s1, Figure S1: ANI heatmap of JC53 and 155 Yersinia phages. Each cell represents the average nucleotide identity (ANI) value between two genomes, with darker shades indicating higher se-quence similarity. The color gradient (scale bar, top right) ranges from low (light) to high (dark) identity values. Distinct clustering patterns are observed among known Yersinia phages, whereas JC53 (highlighted with a red arrow) remains isolated from all other groups, underscoring its low genomic similarity and distinct evolutionary position. Figure S2: Phylogenetic tree based on the complete genome sequences of bacteriophage JC53, 155 Yersinia phages, and 22 *Shigella phages* from the NCBI database. Colored ranges indicate phages infecting *Yersinia pestis* (pink), *Y. enterocolitica* (purple), *Y. pseudotuberculosis* (yellow), and *Shigellaspp*. (blue). Phage JC53 (highlighted in green) is positioned on a separate branch, indicating a distinct evolutionary lineage.
